# P-123. Unlocking Synergy: Exploring Combination Therapies in Whipple's Disease for Enhanced Clinical Efficacy

**DOI:** 10.1093/ofid/ofae631.328

**Published:** 2025-01-29

**Authors:** Hiyam Ghneim, Hashem Haj Ebrahimi, Reema Charles, Tahsin Farid

**Affiliations:** University of Debrecen, Debrecen, Hajdu-Bihar, Hungary; University of Debrecen, Debrecen, Hajdu-Bihar, Hungary; US Food and Drug Administration, Philadelphia, Pennsylvania; US Food & Drug Administration, Richmond, Texas

## Abstract

**Background:**

Whipple's disease (WD) is a rare chronic multisystemic infection that primarily affects the gastrointestinal tract. There are no FDA-approved treatments for WD. Standard treatment involves prolonged antibiotic regimens that can be controversial, especially in cases of relapse or neurological involvement. This study uses CURE ID, an online case reporting platform, to investigate WD cases in the literature, analyze combination therapies currently being used, and identify strategies that improve patient outcomes.

**Figure 1:**
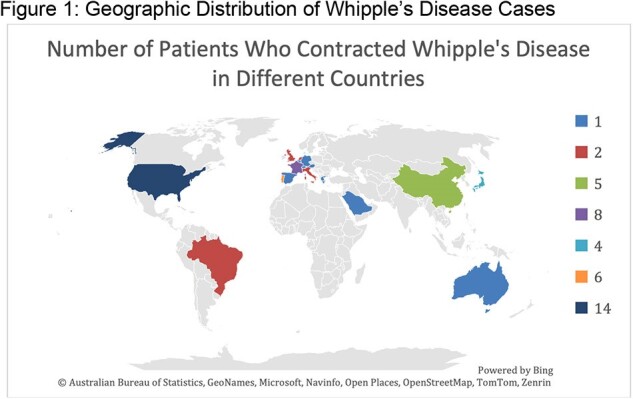
Geographic Distribution of Whipple’s Disease Cases

**Methods:**

We identified WD cases on PubMed and Embase reported between 2019-2024 using relevant keywords, MeSH terms, and Emtree terms. Rayyan.ai was used to screen articles following a PRISMA protocol. Out of 271 identified cases, 84 met inclusion criteria. They were added to CURE ID via the case report form. The aggregated WD data was then analyzed.

**Figure 2:**
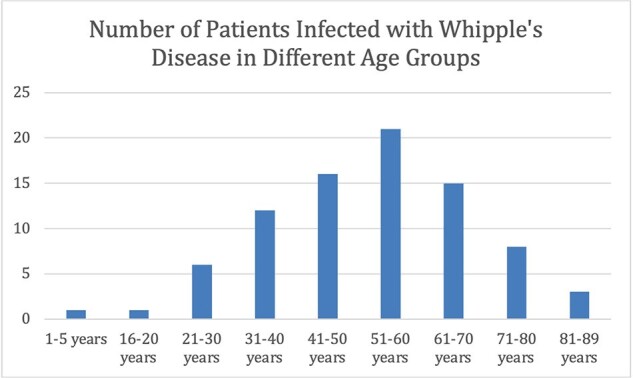
Age Distribution of Whipple’s Disease Patients

**Results:**

74/84 patients improved or recovered (Table1). Figure 1 displays that most cases originated in Europe and North America, followed by a minority in Asia and South America. WD predominantly occurred in males (61/84) and most cases occurred in middle-aged patients (Figure 2). Most cases were associated with favorable outcomes (Table 1). As shown in Figure 3, the most used antibiotic combination was ceftriaxone and TMP-SMX and was associated with the best outcome followed by doxycycline and hydroxychloroquine. Corticosteroids were added in 4 patients to prevent the formation of Immune Reconstitution Inflammatory Syndrome (IRIS), and all cases were associated with a positive result.

**Figure 3:**
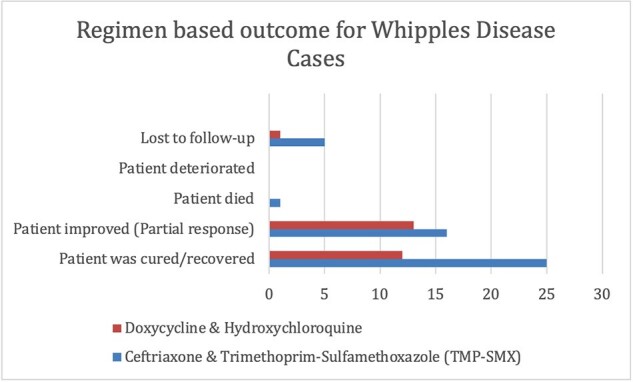
Outcome of the most frequently used treatment regimens

**Conclusion:**

There appears to be a geographic clustering of WD cases as well as higher incidence in males. This needs to be investigated further for explanations for this correlation. It was noted that the majority of the cases occurred in immunocompromised patients. We aim to investigate this further in a full manuscript. The standard treatment of WD is using combination therapy with ceftriaxone and TMP-SMX and this was associated with favorable outcomes. Interestingly, the combination of doxycycline and hydroxychloroquine also emerged as a successful alternative in cases where the preferred combination was not tolerated or failed. We aim to further explore the mechanism of this combination and establish it as a standard alternative regimen.

**Table 1:**
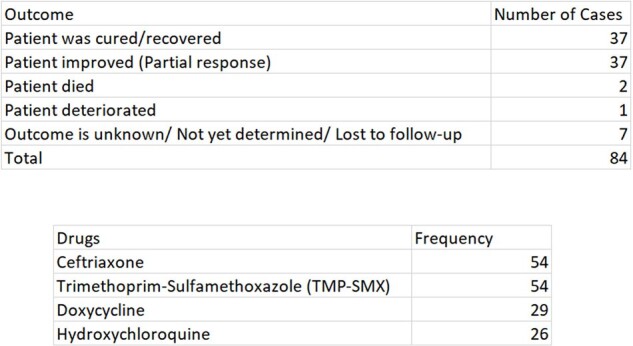
Composite table of patient outcomes and frequency of drugs used.

**Disclosures:**

**All Authors**: No reported disclosures

